# Oral Hobnail Hemangioma of the Maxillary Labial Mucosa: Clinicopathologic Findings of a Rare Case

**DOI:** 10.1155/crid/8935727

**Published:** 2026-08-30

**Authors:** Kevser Dinç, Füsun Yaşar, Murat Çelik

**Affiliations:** ^1^ Department of Oral and Maxillofacial Radiology, Faculty of Dentistry, Selçuk University, Konya, Türkiye, selcuk.edu.tr; ^2^ Department of Pathology, Faculty of Medicine, Selçuk University, Konya, Türkiye, selcuk.edu.tr

**Keywords:** CD34, hobnail hemangioma, immunohistochemistry, oral pathology, oral vascular lesion

## Abstract

Oral hobnail hemangioma is an exceptionally rare benign vascular lesion of the oral cavity. A 57‐year‐old edentulous woman presented for evaluation of discomfort associated with her existing prosthesis, and intraoral examination revealed a bluish‐purple, smooth‐surfaced, asymptomatic nodular lesion located in the anterior maxillary labial mucosa. Clinically, vascular malformation and hemangioma were considered in the differential diagnosis. Histopathological examination revealed dilated superficial vascular channels within the lamina propria, some of which contained erythrocytes, together with characteristic hobnail endothelial cell morphology in the superficial aspect of the lesion. Immunohistochemical analysis demonstrated CD34 positivity and HHV‐8 negativity. Based on the clinical, histopathological, and immunohistochemical findings, a diagnosis of oral hobnail hemangioma was established. Accurate recognition of this rare entity is important because of its clinical and histopathological overlap with other benign, intermediate‐grade, and malignant vascular proliferations, particularly Kaposi sarcoma and hemangioendothelioma variants.

**Clinical Relevance**

Oral hobnail hemangioma is an exceptionally rare vascular lesion that may clinically mimic vascular malformations and low‐grade malignant vascular proliferations, underscoring the need for careful diagnostic correlation to avoid inappropriate management.

## 1. Introduction

Hobnail hemangioma, first described by Santa Cruz and Aronberg in 1988 under the term “targetoid hemosiderotic hemangioma” [1], is an uncommon benign vascular neoplasm histopathologically defined by dilated vascular channels lined by distinctive hobnail‐shaped endothelial cells. Although the lesion predominantly involves the skin of the extremities and trunk, its occurrence within the oral cavity is exceedingly rare [2, 3].

Vascular lesions of the oral cavity encompass a heterogeneous spectrum of hemangiomas and vascular malformations that frequently share overlapping clinical and histopathological features, often leading to diagnostic confusion in clinical practice [4, 5]. Hemangiomas arise from endothelial cell proliferation and predominantly affect the head and neck region, with a recognized female predilection [6, 7]. These lesions typically manifest as asymptomatic bluish or violaceous nodular swellings, most frequently affecting the lips, tongue, buccal mucosa, and palate, whereas jaw involvement remains uncommon [5–7]. Although these lesions are generally asymptomatic, bleeding may occur depending on the lesion size and location. Blanching upon digital pressure is regarded as a characteristic clinical sign, and diagnosis relies largely on patient history together with clinical examination [7].

Accurate recognition of hobnail hemangioma is clinically important, as it may closely mimic other benign, intermediate‐grade, and malignant vascular proliferations, particularly Kaposi sarcoma and hemangioendothelioma variants, both clinically and microscopically, underscoring the need for a comprehensive diagnostic approach. The present report describes the clinical, radiological, histopathological, and immunohistochemical features of a rare case of oral hobnail hemangioma involving the anterior maxillary labial mucosa.

## 2. Case Presentation

**Figure 1:**
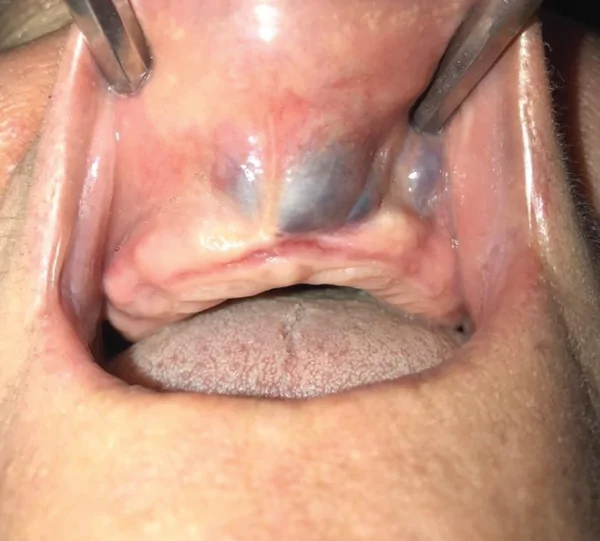
Clinical intraoral photograph demonstrating a bluish‐purple nodular lesion located on the anterior maxillary labial mucosa.

A 57‐year‐old edentulous woman with no significant systemic disease presented to the Department of Oral and Maxillofacial Radiology, Faculty of Dentistry, Selçuk University, for routine clinical examination because of discomfort associated with her existing maxillary prosthesis. Intraoral examination revealed a well‐circumscribed bluish‐purple nodular lesion located on the anterior maxillary labial mucosa (Figure 1). The lesion was smooth‐surfaced, slightly elevated, nonulcerated, and asymptomatic. The patient was unable to recall the exact onset of the lesion and reported no history of pain or bleeding. No cervical or submandibular lymphadenopathy was detected on clinical examination. Digital pressure applied to the lesion resulted in complete blanching, supporting a clinical impression of hemangioma. Based on the clinical presentation, a provisional diagnosis of vascular malformation versus hemangioma was considered, and the patient was subsequently referred for radiographic evaluation and excisional biopsy.

**Figure 2:**
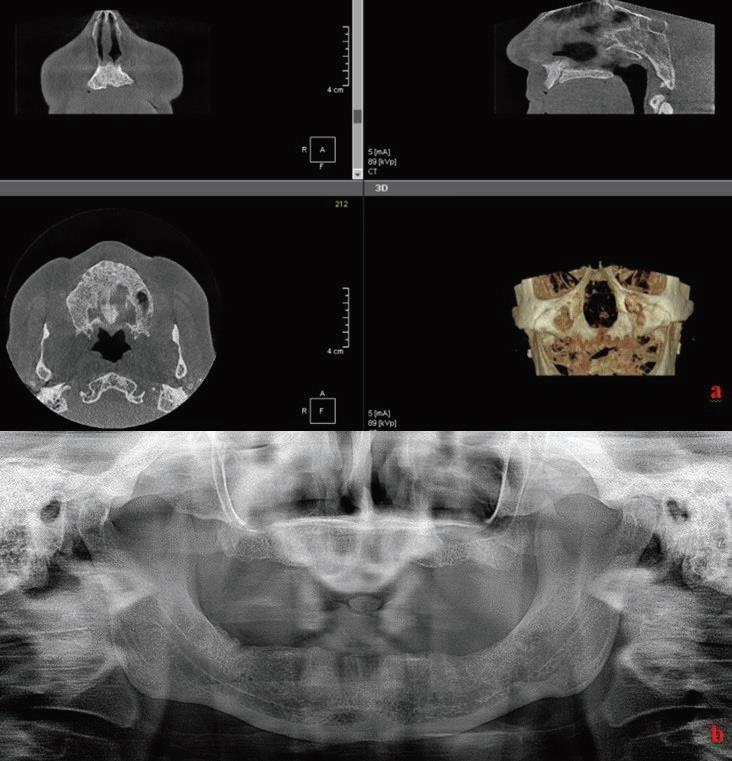
Radiographic examination of the lesion. (a) Cone‐beam computed tomography image demonstrating absence of osseous involvement. (b) Panoramic radiograph showing no radiographic evidence of osseous resorption in the anterior maxillary region.

Panoramic radiography and cone‐beam computed tomography (CBCT) demonstrated no radiographic evidence of osseous resorption, cortical perforation, or destructive bony changes in the anterior maxillary region (Figure 2a,b). Based on the clinical and radiological findings, vascular malformation and hemangioma were considered in the differential diagnosis.

**Figure 3:**
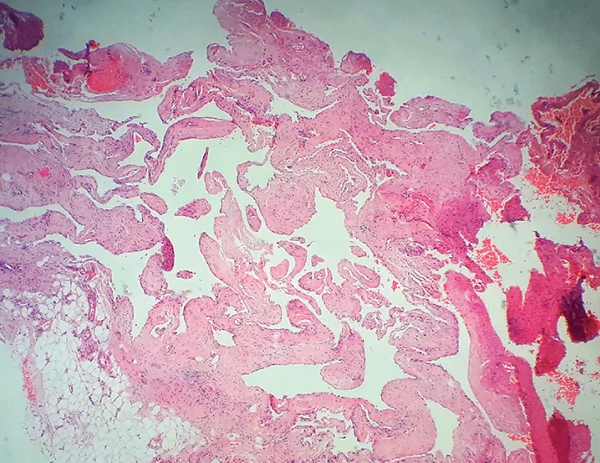
Histopathological examination demonstrating dilated superficial vascular channels within the lamina propria (H&E, ×50).

**Figure 4:**
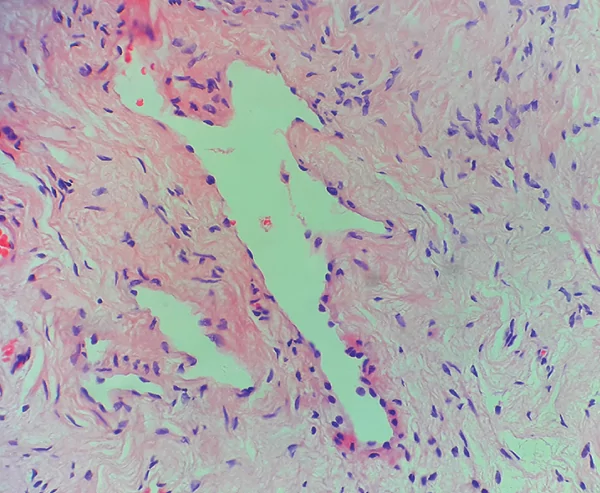
Higher magnification view showing hobnail endothelial cells protruding into the vascular lumen (H&E, ×400).

**Figure 5:**
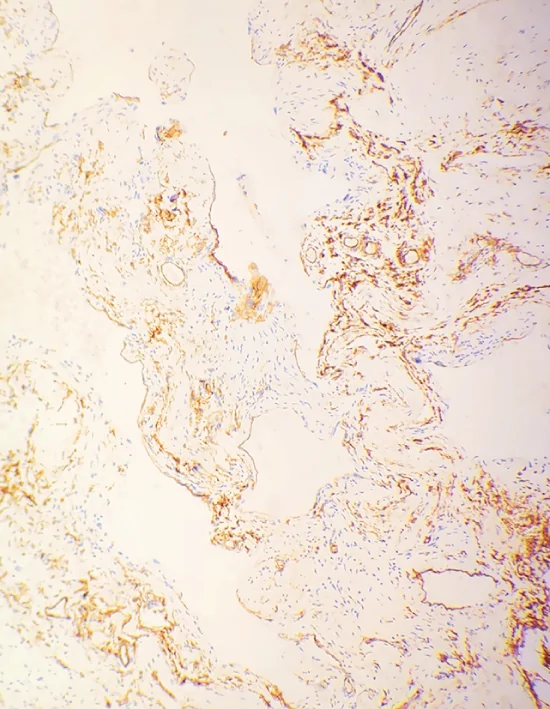
Immunohistochemical staining for CD34 highlights the endothelial lining of the vascular channels (×100).

**Figure 6:**
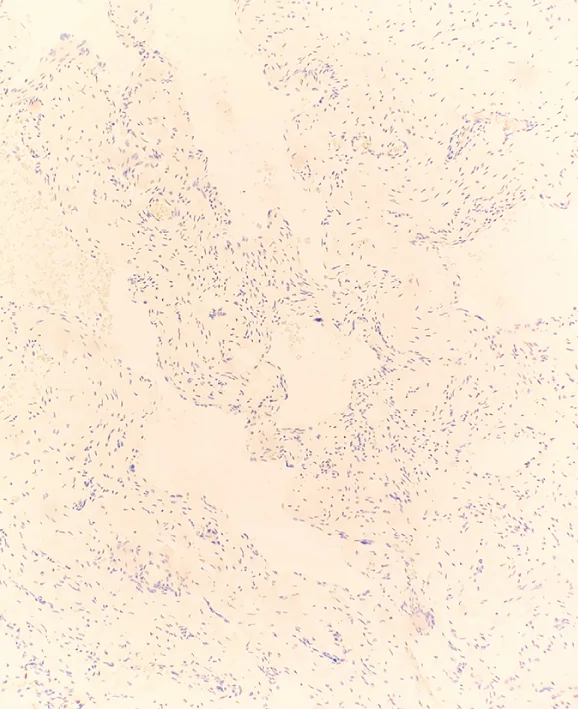
Immunohistochemical staining for HHV‐8 is negative in the lesion (×100).

An excisional biopsy was performed under local anesthesia, and two soft tissue specimens measuring 0.7 × 0.6 × 0.5 and 1 × 0.5 × 0.5 cm were submitted for histopathological examination. Histopathological examination revealed dilated superficial vascular channels within the lamina propria, some of which contained erythrocytes (Figure 3). The superficial vascular spaces were lined by endothelial cells demonstrating characteristic hobnail morphology with focal papillary projections into the vascular lumen (Figure 4). Immunohistochemical analysis showed CD34 positivity in the vascular endothelial cells (Figure 5), whereas HHV‐8 staining was negative (Figure 6). These findings collectively supported the diagnosis of oral hobnail hemangioma. Histopathological and immunohistochemical examinations were performed at the Department of Pathology, Faculty of Medicine, Selçuk University. The patient presented to our department in November 2022, and excisional biopsy was subsequently performed. Postoperative follow‐up examinations conducted at 3 and 6 months after excision revealed no clinical evidence of recurrence, following which the patient was referred to the prosthodontics department for management of her denture‐related complaint. The patient expressed satisfaction with the treatment outcome.

## 3. Discussion

Hobnail hemangioma is characterized by considerable variability in its clinical and histopathological presentation, which can complicate its differentiation from other benign and low‐grade malignant vascular proliferations [2]. Although the lesion most commonly occurs on the skin of the extremities and trunk, involvement of the oral cavity has rarely been reported in the literature [2, 3, 8, 9]. Although hobnail hemangioma is more commonly reported in patients in their 20s and 30s [2, 10], oral involvement is not confined to young adults. Previously reported oral cases include patients aged 73 and 84 years, and the present patient was 57 years old (Table 1).

**Table 1:** Reported cases of oral hobnail hemangioma, including the present case.

Author (year)	Age/sex	Site	Imaging findings	Immunohistochemistry	Treatment	Follow‐up/recurrence
Guillou et al. [11] (1999), Case 2	22/F	Right side of the tongue (infiltrating between superficial skeletal muscle bundles)	None reported	CD31+, CD34+, Factor VIII‐related antigen+, UEA‐1+ (weaker); actin‐positive pericytes only focal and discontinuous around lesional vessels	Excisional biopsy	7 months/no recurrence, no metastasis
Guillou et al. [11] (1999), Case 15	12/M	Left mandibular gingiva, around the premolars and molars	None reported	Not performed	Excision biopsy	48 months/no recurrence, no metastasis
Rashmi et al. [12] (2008)	24/M	Mandibular left gingiva	?	?	Excisional biopsy (as reported by Salasuo et al. [2])	?
Vieira Cury et al. [13] (2009)	38/F	Hard palatal mucosa	?	?	?	?
Hejnold et al. [14] (2012)	73/F	Tongue	?	?	Resection of the nodule	?
Hiremath et al. [8] (2013)	25/M	Mandibular anterior gingiva, extending from the lower labial sulcus to the lingual sulcus (mesial aspect of the right central incisor to the distal aspect of the left canine)	Intraoral periapical radiograph: interdental bone loss between the central incisors, extending to the middle third of the root	CD31 strongly+, Factor VIII strongly+, CD34 moderately+, VEGF−	Surgery (not further specified)	6 months/no recurrence
Alhassani et al. [15] (2018)	53/F	Mandibular gingiva (interdental papillae between the lower right canine and lateral incisor, extending buccolingually)	Radiographs: no evidence of bone loss around the teeth	CD31+, CD34+, Factor VIII+, D2‐40+, HHV‐8−	Surgical excision; recurrence at 5 months, excised a second time	14 months after the second excision/recurrence (after the initial, incomplete excision)
Kunisada et al. [16] (2021)	84/F	Lower surface of the right side of the tongue	?	D2‐40+ and CD34− in the dilated superficial vessels; D2‐40 and/or CD34+ in the deeper slit‐like vessels; few *α*‐SMA‐positive perivascular cells	Total resection under local anesthesia; after recurrence, the mass and surrounding tissue were totally resected	Recurrence 6 months after the initial resection; no signs of recurrence thereafter
Sato et al. [3] (2024)	50/F	Right lateral tongue	MRI: poorly circumscribed low‐signal area (approximately 25 × 7 mm) on T1‐weighted images; poorly circumscribed medium‐to‐high‐signal area on fat‐suppressed T2‐weighted images	CD31+, D2‐40+, ERG+ (nuclear), WT1−	Resection under general anesthesia with a safety margin of approximately 5 mm	24 months/no recurrence
Salasuo et al. [2] (2026)	7/F	Gingiva (upper left canine region, extending buccopalatinally)	Head‐and‐neck MRI and abdominal ultrasound: no abnormality; MRI repeated at the 18‐month control, no recurrence	CD34+ (biphasic staining pattern), GLUT1−, WT1−	Complete excision under general anesthesia	18 months/no recurrence (the lesion had recurred after the initial biopsy)
Present case (2026)	57/F	Maxillary labial mucosa	Panoramic radiography and CBCT: no osseous resorption, cortical perforation or destructive bony change	CD34+, HHV‐8−	Excisional biopsy under local anesthesia	6 months/no recurrence

*Note:* Cases are listed chronologically. Question marks indicate data that were not available in the source consulted. For the cases of Rashmi et al., Vieira Cury et al., and Hejnold et al. only the English abstract was accessible to the present authors; the report of Kunisada et al. is published in Japanese and was assessed from its English abstract and figure legends.

Abbreviations: CBCT, cone‐beam computed tomography; MRI, magnetic resonance imaging; SMA, smooth muscle actin.

To date, only a limited number of oral hobnail hemangiomas have been reported. Clinically, hobnail hemangioma is usually a solitary, small, well‐demarcated lesion whose appearance is nonetheless variable; among the published oral cases, the color has ranged from indistinguishable from the adjacent mucosa to pale blue, blue, or purplish red [2]. Hiremath et al. [8] and Rashmi et al. [12] each described oral hobnail hemangioma with histopathologically confirmed hobnail endothelial morphology; notably, the lesion reported by Hiremath et al. was sessile and exophytic, irregular and cauliflower‐like in shape, and of the same color as the adjacent mucosa rather than bluish‐purple, illustrating that the clinical appearance of this entity is variable and that the bluish‐purple presentation observed in the present case is not universal.

The clinicopathologic features of all previously published oral hobnail hemangiomas, together with the present case, are summarized in Table 1. Notably, imaging findings were reported in only four of the previously published cases, and none of them was evaluated with CBCT. A recent systematic review of the English‐language literature identified seven previously published intraoral cases [2]; two further oral cases [14, 16] and the case reported by Salasuo et al. themselves bring the total to 10 previously published cases (Table 1). These involved the gingiva (*n* = 5), the tongue (*n* = 4), and the palate (*n* = 1), with patient age ranging from 7 to 84 years (median 31.5 years). Notably, none of these previously reported cases involved the labial mucosa, and the present case is the first oral hobnail hemangioma reported at this subsite. This suggests that the diagnosis should remain in the differential regardless of patient age or anatomical subsite within the oral cavity.

Regarding clinical course, previously reported oral hobnail hemangiomas have generally shown a favorable prognosis following complete surgical excision. Recurrence was nevertheless reported in 3 of the 10 previously published cases; in each instance, it followed the initial excision or biopsy and was subsequently controlled by a further, wider excision. Follow‐up, where reported, ranged from 6 to 48 months (median 16 months) (Table 1). In the present case, clinical follow‐up at 3 and 6 months demonstrated no evidence of recurrence, consistent with the generally indolent course reported for this entity; however, given the relatively short observation period, continued clinical surveillance remains warranted.

Previous immunohistochemical studies have demonstrated positivity for endothelial markers such as CD31 and CD34 in hobnail hemangioma lesions, supporting their vascular endothelial origin [17]. Hejnold et al. [17] additionally reported that the superficial portions of hobnail hemangioma characteristically contain dilated vascular spaces lined by plump endothelial cells with hobnail morphology, findings that were also observed in the present case. Comparable endothelial morphology and CD34 positivity have also been described in oral vascular lesions with epithelioid vascular differentiation and oral epithelioid hemangioma cases [18, 19]. The histogenesis of hobnail hemangioma remains debated: positivity for the lymphatic endothelial marker D2‐40 has led some authors to regard the lesion as lymphatic in origin, or as a lymphatic malformation rather than a neoplasm, whereas others have found consistent expression of blood‐vascular markers with absent or only focal D2‐40 staining and have argued against a lymphatic origin [17, 20–23].

In the present case, CD34 positivity and the absence of significant cytologic atypia supported the benign vascular nature of the lesion. CD34 confirms the vascular endothelial lineage of the lesional cells [17], thereby distinguishing hobnail hemangioma from nonvascular mimics, whereas HHV‐8 negativity is of particular diagnostic value in this context, as it excludes Kaposi sarcoma [24]—the most important malignant mimicker of hobnail hemangioma in the oral cavity—given their overlapping bluish‐purple clinical appearance. Unlike conventional hemangiomas—which arise through endothelial proliferation and typically involute over several years—and vascular malformations—which grow slowly throughout life and do not involute [5, 7]—hobnail hemangioma is a persistent but self‐limited lesion adequately treated by simple excision, with little tendency to recur [2]. Immunohistochemically, CD34 showed a biphasic staining pattern in the case reported by Salasuo et al. [2], with GLUT‐1 and WT1 negative—the former contrasting with infantile hemangioma, in which GLUT‐1 is expressed in over 95% of cases [25]. A limitation of the present case is that immunohistochemical evaluation was restricted to CD34 and HHV‐8; additional endothelial markers (e.g., CD31, ERG, or FLI1) or lymphatic‐associated markers (e.g., D2‐40/podoplanin) were not performed. Given the ongoing debate regarding the vascular versus lymphatic histogenesis of hobnail hemangioma [20–23], a broader immunohistochemical panel could have provided further insight into the lineage of the lesional endothelial cells in this case.

Hobnail hemangioma may clinically mimic several pigmented and vascular lesions, including pigmented nevus, malignant melanoma, Kaposi sarcoma, conventional hemangioma, solitary angiokeratoma, and mucocele; the key clinical features distinguishing these entities are summarized in Table 2 [5–7, 26–29, 31, 32].

**Table 2:** Clinical differential diagnosis of hobnail hemangioma.

Differential diagnosis	Clinical features	Distinguishing feature from hobnail hemangioma
Pigmented nevus [26]	Brown or blue lesion; junctional nevi are flat and dark brown, whereas intramucosal and compound nevi are light brown and dome‐shaped; the blue nevus derives its color from melanocytes situated deep in the connective tissue	Does not blanch with digital pressure, reflecting its melanocytic (nonvascular) origin.
Malignant melanoma [26, 27]	Irregular, asymmetric borders; variable pigmentation (dark brown to bluish‐black), occasionally gray, purple, or red due to ulceration; most frequently affects the hard palate and maxillary gingiva	Does not blanch with digital pressure; frequently shows ulceration, rapid growth, and irregular color variegation.
Kaposi sarcoma [28–30]	Bluish‐purple nodular or plaque‐like lesion of the oral mucosa; associated with immunosuppression	HHV‐8 positive; histologically shows spindle‐cell proliferation with cellular/nuclear atypia.
Conventional (capillary/cavernous) hemangioma [5–7, 31]	Bluish‐purple, compressible, soft nodular swelling	Clinically indistinguishable from hobnail hemangioma, as both blanch completely with digital pressure; a feeder vessel on Doppler ultrasonography may favor a conventional hemangioma, but definitive distinction requires histopathological examination.
Solitary angiokeratoma [32]	Reddish to bluish papule, often with a hyperkeratotic or verrucous surface and petechial hemorrhages; may bleed with trauma	Overlying epithelial hyperplasia with hyperkeratosis and acanthosis over dilated vascular channels, features absent in hobnail hemangioma.
Mucocele [31]	Bluish, fluctuant, mucin‐filled swelling; most common on the lower labial mucosa; history of trauma common	Aspiration yields mucin rather than blood; no feeder vessel on ultrasonography.

The histopathological differential diagnosis of hobnail hemangioma is broad, encompassing benign, intermediate‐grade, and malignant vascular proliferations, as well as lymphatic and fibroblastic lesions. Specifically, it includes in particular Kaposi sarcoma, retiform hemangioendothelioma, benign lymphangioendothelioma, and well‐differentiated angiosarcoma [12]. The key microscopic features distinguishing these entities from hobnail hemangioma are summarized in Table 3 [2, 5, 7, 11, 24, 25, 28–36].

**Table 3:** Histopathological differential diagnosis of hobnail hemangioma, grouped by biological behavior.

Differential diagnosis	Histopathological features	Distinguishing feature from hobnail hemangioma
Capillary hemangioma [5, 7, 11]	A benign vascular tumor that develops through endothelial cell proliferation; the superficial subgroup, consisting of small capillary bodies organized in a lobular arrangement.	A lobular proliferation of capillary‐sized vessels; lacks the hobnail endothelial cytomorphology, the biphasic superficial‐to‐deep architecture and the collagen‐dissecting deep component of hobnail hemangioma.
Cavernous hemangioma [5, 7, 11]	The deep subgroup of hemangioma, consisting of wide, dilated vessels that may reach a large size; phleboliths (calcified thrombi) may form within its veins, venules, and sinusoidal vessels.	Uniformly wide and dilated vascular spaces; lacks hobnail cytomorphology, the biphasic growth pattern and the collagen‐dissecting deep component of hobnail hemangioma.
Vascular malformation [5, 11]	Characterized by hypocellularity and vascular channels lined by flat, mature endothelium; results from abnormal vascular or lymphatic morphogenesis rather than endothelial proliferation.	The endothelium is flat and mature without hobnail cytomorphology, and the lesion is hypocellular; the biphasic architecture and collagen‐dissecting deep component of hobnail hemangioma are absent.
Spindle cell hemangioma [30]	Biphasic architecture with cavernous vascular spaces containing thrombi, alternating with fascicles of spindle cells and clear endothelial vacuoles resembling adipocytes; numerous extravasated erythrocytes.	A spindle‐cell proliferation with vacuolated cells predominates, and hobnail endothelial cytomorphology is absent; ERG and CD31 are positive in endothelial cells but negative in the spindle cells.
Pyogenic granuloma [2, 25]	Lobules of capillary‐sized vessels separated by fibrous septa, resembling granulation tissue; inflammation is often conspicuous and may be acute, chronic or both, and the surface may be ulcerated.	Hobnail hemangioma lacks the lobules of capillary‐sized vessels separated by fibrous septa that characterize pyogenic granuloma; notably, in two of the reported oral cases the lesion was initially diagnosed as pyogenic granuloma and reclassified only after recurrence.
Solitary angiokeratoma [11, 32]	Hyperparakeratosis and acanthosis of the overlying epithelium with papillary projections, together with dilated subepithelial vessels filled with erythrocytes.	Defined by the reactive hyperplasia of the overlying epithelium; the hobnail endothelial cytomorphology, biphasic architecture, and collagen‐dissecting deep component of hobnail hemangioma are absent.
Lymphangioma [25]	Dilated lymphatic channels lined by a thin endothelium and intimately associated with the overlying epithelium; the spaces contain proteinaceous fluid and scant lymphocytes.	A hamartomatous malformation of lymphatic channels rather than a neoplasm; the lining endothelium is thin and lacks hobnail cytomorphology. The lymphatic content assists the distinction, although erythrocytes may enter the channels following trauma and their presence does not exclude the diagnosis. CD31 and CD34 label the endothelium of all vascular lesions, whereas only lymphatic endothelium reacts with D2‐40.
Benign lymphangioendothelioma [11]	Thin‐walled, irregularly branching vascular channels dissecting the stroma, relatively uniform throughout the lesion.	Displays a relatively uniform microscopic appearance at low magnification, whereas hobnail hemangioma characteristically demonstrates a biphasic growth pattern; the hobnail cytomorphology is also less pronounced, and most of the vascular spaces are devoid of erythrocytes.
Angiofibroma [11, 31]	Fibrous connective tissue containing abundant collagen fibers and proliferating fibroblasts, with dilated blood vessels and sinusoidal spaces lined by endothelial cells.	A predominantly fibroblastic and stromal proliferation; the hobnail endothelial cytomorphology and biphasic architecture of hobnail hemangioma are absent. Stromal cells are strongly vimentin positive and generally negative for smooth muscle actin.
Solitary fibrous tumor (hemangiopericytoma) [33]	Variably patternless proliferation of spindle to ovoid cells in a collagenous to myxoid stroma, with branching blood vessels showing perivascular hyalinization; hypocellular and hypercellular areas alternate.	A fibroblastic rather than endothelial neoplasm, classified by the WHO as a borderline/low‐grade soft tissue tumor; the neoplastic cells do not line the vascular channels and hobnail cytomorphology is absent. CD34 is positive in both entities, but nuclear STAT6 expression, reflecting the NAB2‐STAT6 fusion, distinguishes solitary fibrous tumor from its histological mimics.
Epithelioid hemangioendothelioma [34, 35]	Diffuse proliferation of epithelioid endothelial cells with hyperchromatic nuclei and intracytoplasmic vacuoles, some containing erythrocytes, within numerous vascular spaces.	Endothelial cells are epithelioid rather than hobnail in cytomorphology and show intracytoplasmic vacuolization; an intermediate‐grade neoplasm with an indolent course that rarely metastasizes. CD31 and Factor VIII are diffusely positive with only weak CD34 expression, contrasting with CD34 positivity of the present case.
Retiform hemangioendothelioma [36]	Elongated, branching, thin‐walled vascular channels arranged in a pattern resembling the rete testis, lined by monomorphic hobnail endothelial cells.	Shares hobnail endothelial cytomorphology with hobnail hemangioma, but is defined by its distinctive arborising retiform vascular architecture.
Kaposi sarcoma [24, 26, 28–30]	A proliferation of spindle‐shaped cells surrounding poorly formed vascular spaces or slits, with numerous extravasated red blood cells; associated with HHV‐8 infection.	Shows a more infiltrative growth pattern, higher mitotic activity, and cellular/nuclear atypia; the neoplastic component is a spindle‐cell proliferation rather than hobnail endothelium. HHV‐8 is positive, whereas it is consistently absent in hobnail hemangioma, as in the present case.
Well‐differentiated angiosarcoma [11]	Anastomosing vascular channels dissecting the stroma, showing endothelial multilayering and tufting, cytologic atypia, and mitotic activity.	Hobnail hemangioma may display a collagen‐dissecting, pseudoangiosarcomatous pattern in its deeper portions, but consistently lacks endothelial multilayering or tufting and shows minimal or absent cytologic atypia without mitoses.

The diagnostic value of HHV‐8 negativity extends to other pseudosarcomatous vascular lesions capable of mimicking Kaposi sarcoma; Mani et al. [30] similarly reported an intraoral spindle cell hemangioma initially suspected to represent Kaposi sarcoma based on histopathological features alone, in which HHV‐8 negativity—together with ERG and CD31 positivity—was likewise essential in establishing a benign vascular diagnosis, reinforcing the diagnostic value of HHV‐8 immunonegativity across histologically overlapping vascular entities.

Epithelioid hemangioendothelioma represents another intermediate‐grade vascular neoplasm within the differential, characterized by epithelioid rather than hobnail‐shaped endothelial cells with intracytoplasmic vacuolization [35]. Bajpai and Pardhe [34] reported a rare intraoral case involving the tongue, in which the diagnosis was supported by diffuse CD31 and Factor VIII positivity together with only weak CD34 expression, the latter contrasting with CD34 positivity observed in the present case. In contrast, well‐differentiated angiosarcoma is characterized by anastomosing vascular channels showing endothelial multilayering and cytologic atypia. Although hobnail hemangioma may exhibit a collagen‐dissecting, pseudoangiosarcomatous pattern in its deeper portions, Guillou et al. [11] demonstrated that it consistently lacks endothelial multilayering or tufting and shows minimal or absent cytologic atypia without mitotic activity, features that reliably separate it from well‐differentiated angiosarcoma. Complete surgical excision is regarded as the treatment of choice for oral hobnail hemangioma and the prognosis is excellent. Salasuo et al. [2] nevertheless emphasize that excision should be performed with adequate margins in order to avoid recurrence. Overall, the present report is limited by its single‐case design, restricted immunohistochemical panel, and relatively short follow‐up period; given the scarcity of previously published oral hobnail hemangioma cases, additional reports with extended long‐term follow‐up are warranted to further clarify the recurrence potential and optimal management of this entity.

## 4. Conclusion

Oral hobnail hemangioma is an exceptionally rare vascular lesion that may clinically mimic other benign and malignant vascular proliferations, as illustrated by the present case—to our knowledge, the first reported instance of this entity involving the labial mucosa. This underscores that the diagnosis should remain in the differential regardless of the anatomical subsite involved. As clinical and radiological findings are often nonspecific, definitive diagnosis relies primarily on histopathological evaluation, highlighting the importance of accurate diagnostic work‐up to guide appropriate, conservative management. Awareness of this entity is essential to prevent overdiagnosis as low‐grade malignant vascular neoplasms and to avoid unnecessary aggressive treatment.

## Author Contributions

**Kevser Dinç:** conceptualization, investigation (clinical and radiological examination), writing – original draft, writing – review and editing. **Füsun Yaşar:** conceptualization, investigation (clinical and radiological examination), supervision, writing – review and editing. **Murat Çelik:** investigation (histopathological evaluation), methodology, writing – review and editing.

## Funding

No funding was received for this manuscript.

## Disclosure

All authors have read and approved the final version of the manuscript. Kevser Dinç (corresponding author) had full access to all of the data in this study and takes complete responsibility for the integrity of the data and the accuracy of the data analysis.

## Ethics Statement

As a single, retrospective, anonymized case report involving no experimental intervention beyond routine clinical care, formal ethics committee approval was not required, in accordance with institutional guidelines and the Declaration of Helsinki. Written informed consent for publication of this case report and the accompanying clinical, radiological, and histopathological images was obtained from the patient, in accordance with ICMJE recommendations.

## Conflicts of Interest

The authors declare no conflicts of interest.

## Data Availability

The data that support the findings of this study are available from the corresponding author upon reasonable request.
